# Mischievous Misstatements

**Published:** 1887-03-12

**Authors:** 


### MISCHIEVOUS MISSTATEMENTS:

A WORD OF COUNSEL TO THE CITY COMPANIES.

ONE of the least well-managed of metropolitan hospitals recently received a very large grant from a City Company. We understand that the mover of the grant gave, amongst others, the following reasons for urging the Court to show extraordinary liberality to this particular institution:—"Hospital Sunday was everywhere a failure because it not only lessened the aggregate amount of the income received by the hospitals as a whole, but it destroyed the source from which that income was originally supplied. People who, before the institution of Hospital Sunday, gave pounds to hospitals, now gave pence, or more probably buttons only, in the offertory bag once a year. So great indeed was the failure of Hospital Sunday, and so serious was the injury which it did to hospitals, that the late Dr. Wakley expressed the deepest regret that he had ever been associated with the Hospital Sunday movement, because he had lived long enough to see that it had resulted in a considerable loss of income to the hospitals, much of which was formerly derived from channels which were, through its establishment and organisation, now closed."

Much interest and sympathy was awakened by so stirring an appeal, though it was founded upon monstrous untruths, and a very large grant was unanimously voted to one particular hospital. We say monstrous untruths, because it cannot be too often repeated that Hospital Sunday in London has resulted, not only in no loss of revenue to the hospitals, but in a clear additional income of upwards of £20,000 per annum, when the charitable receipts in 1885 are compared with those received *pro rata* from the population in the year preceding that in which Hospital Sunday was instituted. In other words, Hospital Sunday in London has produced a clear gain to the hospitals equal to an additional contribution of upwards of three-farthings per head of the entire population.

*The Lancet* will no doubt deal with the statement concerning its late Editor, but we may point out that the late Dr. Wakley not only believed in but loved Hospital Sunday, and that on his deathbed he showed the greatest concern for the increased success of the collections which were proceeding at the time. More than this, it must be remembered, that in each of the last two years of his life he gave £1,000 to the hospitals on Hospital Sunday, in the hope of encouraging others to go and do likewise. The Hospital Sunday and Hospital Saturday movements deserve well of the public and of the supporters of hospitals. Their work is invaluable, if the voluntary principle of supporting hospitals is to be maintained in this country. In these circumstances ridiculous misstatements like those we have here referred to, whether made intentionally or not, demand the fullest exposure and the deepest censure from every honest citizen.

We do not know if the authorities of the various City Companies are as yet aware even of the existence of THE HOSPITAL. This is a busy age, and people do not readily realise the existence of any new undertaking. Indeed, a nurse in a large London hospital writes to us this week to say that she had only just seen THE HOSPITAL for the first time, although copies are regularly taken, not only by the provincial hospitals, but by the London hospitals also, to some of which hundreds of copies are supplied every week. Whatever may be said against the City Companies, no one can deny that they have been the props, and in many cases the backbones, which have sustained the voluntary hospitals of the metropolis year by year. In times of great financial difficulty, in periods of distress when new buildings had to be erected, or enlargements and alterations made; whenever convalescent patients have to be sent to the seaside, and to be there cared for, and often clothed, there has the munificence of the City Companies always been at hand to sustain and help and encourage those responsible for the conduct and maintenance of the metropolitan hospitals. We believe it would astonish the most hostile critic of these Corporations could he be made to realise the vast sums they have freely given to the support of hospitals, and the inestimable benefit derived by the poor from the expenditure of this money.

At the same time, each Court of Assistants has an opportunity still for conferring yet greater benefits upon hospitals, by becoming acquainted with their management, and so being in a position to give to these charities with discrimination and wisdom. If certain members of the Court of Assistants of each Company once realised that the particular hospital under discussion cost 100 per cent. more per bed than another institution for which a grant was applied for on the same day, instead of granting, say £500 to the ill-managed and £50 to the more efficient of the two, we believe, whatever influences might be brought to bear, the majority would act upon the facts. This would obviate decisions which at the present time make the amount of the grants too much dependent upon the influence which is brought to bear by the proposer of the resolution rather than on the deserts of a charity alone. If each City Company would keep a file of THE HOSPITAL, and have it bound up in half-yearly volumes with an index for easy reference, it would always have at hand precise information of an accurate character concerning all the hospitals, which could not fail to interest many members, and to enable all to exercise influence in the direction of making the merits or demerits of a particular institution, the basis for determining whether any, and what grant should be made from the funds of the Company to its support.

We know that the sceptic will say these are counsels of perfection which will never be practically accepted or acted upon. But we do not believe this to be the case, because we feel that an absence of valuable information renders it impossible for the Courts of the City Companies to always decide on the merits of the institutions which apply to them for aid. Knowledge is power, and as we can now supply the Courts and Officers of City Companies with the knowledge they have never yet had within reach, we believe they, for their part, will determine to use the power for good which is placed in their hands by the publication of THE HOSPITAL.

